# Illinois State Board of Health and the Eclectic Medical Institute of Cincinnati

**Published:** 1880-01

**Authors:** 


Illinois State Board of Health and the Eclectic Medical Institute of Cincinnati, Ohio.—At a meeting of the Illinois State Board of Health, held at the Grand Pacific Hotel, Chicago, on Thursday, Dec 11th, the status of the Eclectic Medical Institute was discussed. Since last June the diplomas of this school were not recognized by the board, owing to the fact that it has two courses that count in graduation, in one year, which is contrary to a regulation of the board.

The institute was represented by Boutell and Waterman, of Chicago, as attorneys, and Drs. Scudder and Howe, Professors, of Cincinnati. The State was represented by the attorney-general.

The rule of the board, adopted in 1877, requiring each college to have but one graduating course a year, or its graduates would not be permitted to practice in this State, was gone over in all its phases. No result was arrived at until the evening session, when the following was submitted:

The Eclectic Medical Institute, of Cincinnati, represented here by Drs. Scudder and Howe, agree to abide by the propositions advanced in the rules of the said institute, which are, that three years of medical study are required before graduation, and two courses of lectures of twenty weeks each, and a period of sixteen months at least being occupied by said courses or collegiate instruction, and that the officers of the institute will give certificates to that effect to graduates of said institute, and that diplomas shall only be issued in June.

A. J. HOWE,
JOHN M. SCUDDER.

After deliberation, the following was passed:

*Resolved*, That in view of the foregoing, the diplomas of the Eclectic Medical Institute, of Cincinnati, will be recognized by this board.

The next meeting of the board will be held in Springfield, Jan. 8th, 1880.

---

THE selection, which appears on another page, entitled: "Is Phthisis Self-limiting?" by Dr. Wm. Porter, of St. Louis, is taken from the *St. Louis Medical and Surgical Journal.* The article was set up by us from advance proof-pages, and we only learn, as we go to press, the name of the journal in which the article appears as original matter.

